# Absolute Lymphocyte Count Predicts Bypass Surgery Outcomes in Patients with Chronic Limb-Threatening Ischemia

**DOI:** 10.3400/avd.oa.23-00088

**Published:** 2024-04-10

**Authors:** Satoshi Yamamoto, Takuya Hashimoto, Masaya Sano, Masaru Kimura, Osamu Sato, Juno Deguchi

**Affiliations:** 1 Department of Vascular Surgery, Saitama Medical Center, Saitama Medical University, Kawagoe, Saitama, Japan; 2 Department of Cardiovascular Surgery, Ome Medical Center, Ome, Tokyo, Japan

**Keywords:** chronic limb-threatening ischemia, bypass surgery, absolute lymphocyte count

## Abstract

**Objectives**: The aim of this study was to evaluate the relationship between absolute lymphocyte count (ALC) and outcomes of infrainguinal bypass surgery for chronic limb-threatening ischemia (CLTI).

**Methods**: From 2004 to 2020, 209 limbs of 189 patients who underwent infrainguinal bypass surgery for CLTI and whose ALCs were available were included. Patients with survival >2 years and limb salvage >2 years were considered discriminant groups, and an ALC cut-off value was calculated. The relationship between preoperative ALC and outcomes was evaluated.

**Results**: Survivorship of the higher ALC group was significantly higher than that of the lower ALC group (cut-off value 1030/μL, p = 0.0009). The limb salvage rate of the higher ALC group was significantly higher than that of the lower ALC group (cut-off value 1260/μL, p = 0.0081). In the dialysis patient group (103 limbs), the limb salvage rate of the higher ALC group was significantly higher than that of the lower ALC group (cut-off value 1170/μL, p = 0.026). ALC was independently associated with limb loss in multivariate analysis.

**Conclusion**: ALC is promising as a predictor of outcomes after bypass surgery in CLTI. In particular, ALC is expected to be useful for limb prognosis in hemodialysis patients.

## Introduction

In treating chronic limb-threatening ischemia (CLTI), it is important to consider patient life expectancy to determine the best treatment for limb salvage.^1^^–^^3)^ Previous studies have proposed several models to predict life expectancy and limb salvage in the treatment of CLTI.^4^^–^^8)^ However, a simple and practical predictive method has yet to be established, and there is room for improvement of extant prediction models for several reasons peculiar to CLTI: (1) the diversity of comorbid diseases and the complexity of pathological conditions; (2) two methods of revascularization for ischemia, bypass surgery, and endovascular treatment; (3) advances in endovascular treatment; and (4) institutional or national differences in backgrounds and treatment policy. In particular, patients undergoing hemodialysis tend to have the worst prognosis among patients with CLTI, and it is difficult to predict outcomes after revascularization in patients undergoing hemodialysis.^9^^,^^10)^ In Japan, approximately 50% of patients undergoing hemodialysis need revascularization treatment for CLTI.^11^^,^^12)^ It is still necessary to search for an accurate and straightforward indicator to improve predictive methods for CLTI prognosis.

Poor nutritional status and hemodialysis are among the most common risk factors in predicting survival and limb prognosis in patients with CLTI.^9)^ There is a strong correlation between nutritional status and immunological capacity.^13)^ Factors to assess patient nutritional and immune status include serum albumin and body mass index, and multi-item nutritional evaluation measures, such as the Geriatric Nutritional Risk Index (GNRI) and controlling nutritional status (CONUT) score.^9)^ Among these, absolute lymphocyte count (ALC) is supposed to be one of the most objective and simple indicators because it is little affected by short-term inflammation and preoperative treatment. In the treatment of CLTI, ALC is a statistically significant predictor of amputation-free survival, as described in the univariate analysis in the PREVENT III derivation set.^14)^ However, few reports are available on ALC in the treatment of CLTI, and the usefulness of ALC remains to be clarified.

When considering a revascularization method for CLTI, it is important to predict the outcomes of bypass surgery and endovascular treatment. We previously reported that the CONUT score, calculated from ALC, serum albumin level, and total cholesterol level, may be predictors of survival and limb salvage in patients undergoing bypass surgery for CLTI.^15)^ However, the serum albumin level fluctuates due to fluid infusion and hepatic function, and the total cholesterol level is affected by diet and medication. On the other hand, ALC is supposed to be stable, irrespective of those factors. In this study, we examined patients who underwent infrainguinal bypass surgery for CLTI in order to clarify the usefulness of ALC in predicting survival and limb salvage.

## Subjects and Methods

### Patients

From 2004 to 2020, 209 limbs of 189 patients who underwent infrainguinal bypass surgery for CLTI with tissue loss due to atherosclerosis obliterans and whose ALCs could be calculated were included in the study. Values of each measurement were the most recent values measured before the revascularization procedure was performed (in principle, within one week).

This study was conducted following ethical guidelines for medical research involving human subjects and was approved by the Institutional Review Board of Saitama Medical Center (approval no. 2008).

### Surgical strategy

As a surgical strategy, if a patient was judged to be operable as per preoperative systemic evaluation, infrainguinal revascularization was performed for limb salvage. Preoperative, contrast-enhanced, computed tomography (CT), angiography, and ultrasonography were performed, and bypass surgery was considered to ensure in-line flow to the foot. Patients without an appropriate arterial anastomosis or an autologous vein graft for distal bypass were deemed ineligible for limb bypass. Patients with necrosis or infection extending above the heel were excluded because they were not eligible for limb salvage bypass surgery. In addition, patients subjected to incomplete revascularization were also excluded. In the postoperative period, graft patency was confirmed by palpation, Doppler auscultation, ultrasonography (duplex scan), or CT scans recommended at specific intervals: one week, one month, 3 and 6 months, one year, and every 6–12 months after that.

### Endpoints

Relationships between preoperative ALC and patient background, clinical findings, survival rate, and limb salvage rate were retrospectively evaluated.

### Statistical analysis

Data were presented as means and standard deviations (SDs). Student’s t-test or Chi-square/Fisher’s exact test was used for statistical analysis. Patients with survival of >2 years and limb salvage were considered discriminant groups. The Youden Index and the receiver operating characteristic curve were used to calculate the ALC cut-off value. Survival and limb salvage were compared. The Kaplan–Meier method was used to construct survival and limb salvage curves, and the difference between the two groups was determined using the log-rank test. Cox regression analysis was performed on the ALC and factors contributing to survival or limb salvage. The threshold for statistical significance was set at p <0.05. Similarly, outcomes were evaluated, and survival and limb salvage were compared in the dialysis patient group.

## Results

### Patient characteristics

Patient backgrounds are presented in **Table 1**. The mean ALC was 1282/μL (ALC reference values, 1500–3200/μL). Ninety-one patients with 103 affected limbs (49%) underwent hemodialysis. ALC was lower in the dialysis patient group than in the nondialysis patient group (mean ALC: 1017/μL vs. 1539/μL, p <0.0001), although there was no difference in the white blood cell and neutrophil counts between the two groups.

**Table:** Table 1 Demographic characteristics and preoperative risk factors

	All (209 limbs)	Dialysis patient group (103 limbs)	Nondialysis patient group (106 limbs)	p Value
Age (years), mean ± SD	72.7 ± 8.6	71.7 ± 7.7	73.7 ± 9.2	0.082
Male/female	156/53	81/22	75/31	0.21
Body mass index (kg/m^2^)	21.7 ± 3.5	21.7 ± 3.2	21.8 ± 3.7	0.85
Smoking history	155 (74%)	76 (74%)	79 (75%)	1.0
Current smoking	55 (35%)	19 (19%)	36 (34%)	
Ex-smoking	100 (65%)	57 (55%)	43 (41%)	
Comorbid diseases				
Hypertension, n (%)	164 (78%)	87 (84%)	77 (73%)	0.044
Dyslipidemia, n (%)	70 (33%)	30 (29%)	40 (38%)	0.24
Diabetes mellitus, n (%)	157 (75%)	89 (86%)	68 (64%)	0.0002
Coronary artery disease, n (%)	68 (33%)	40 (39%)	28 (26%)	0.076
Chronic heart failure, n (%)	32 (15%)	19 (18%)	13 (12%)	0.25
Cerebrovascular disease, n (%)	57 (27%)	33 (32%)	24 (23%)	0.16
Medications				
Anticoagulant drug use	42 (33%)	21 (20%)	21 (20%)	1.0
Antiplatelet drug use	175 (84%)	93 (90%)	82 (77%)	0.014
Statin use	59 (28%)	23 (22%)	36 (34%)	0.067
Ambulatory status at registration				
Nonambulatory	118 (56%)	61 (59%)	57 (54%)	0.49
WIfI classification				0.47
Grade 1	2 (1%)	1 (1%)	1 (1%)	
Grade 2	6 (3%)	2 (2%)	4 (4%)	
Grade 3	44 (21%)	17 (16%)	27 (25%)	
Grade 4	157 (75%)	83 (81%)	74 (70%)	
Contralateral limb status				
CLTI	90 (43%)	57 (55%)	33 (31%)	0.0005
Laboratory data				
White blood cells (count/μL), mean ± SD	8502 ± 3285	8430 ± 2908	8572 ± 3626	0.75
Neutrophil (count/μL), mean ± SD	6361 ± 3134	6482 ± 2709	6243 ± 3507	0.58
Lymphocytes = ALC (count/μL), mean ± SD	1282 ± 637	1017 ± 454	1539 ± 684	<0.0001
Hemoglobin (g/dL), mean ± SD	10.63 ± 1.86	9.78 ± 1.34	11.46 ± 1.92	<0.0001
Serum albumin (g/dL), mean ± SD	3.14 ± 0.59	3.00 ± 0.52	3.26 ± 0.62	0.0013
C-reactive protein (mg/dL), mean ± SD (range, median)	3.79 ± 4.89 (0–24.9, 1.6)	4.5 ± 5.3 (0.0–24.9, 2.5)	3.1 ± 4.3 (0.0−22.5, 1.2)	0.033
Total cholesterol (mg/dL), mean ± SD	161.2 ± 44.6 (n = 198)	144.9 ± 38.1 (n = 96)	176.4 ± 45.0 (n = 102)	<0.0001
Distal anastomosis site in infrainguinal arterial reconstruction				
Popliteal artery above the knee, n (%)	34 (16%)	17 (16%)	17 (16%)	
Popliteal artery below the knee, n (%)	32 (15%)	15 (15%)	17 (16%)	
Tibioperoneal trunk, n (%)	6 (3%)	1 (1%)	5 (4%)	
Anterior tibial artery, n (%)	34 (16%)	17 (16%)	17 (16%)	
Posterior tibial artery, n (%)	39 (19%)	18 (18%)	21 (20%)	
Peroneal artery, n (%)	16 (8%)	6 (6%)	10 (10%)	
Dorsal pedis artery, n (%)	35 (17%)	26 (25%)	9 (8%)	
Plantar artery, n (%)	13 (6%)	3 (3%)	10 (10%)	

WIfI: Wound, Ischemia, and foot Infection; CLTI: chronic limb-threatening ischemia; ALC: absolute lymphocyte count

### Causes of death

The causes of death are presented in **Table 2**. Cardiovascular events were the most common cause of death, and sepsis was the second. Among dialysis patients, cardiovascular events were the most common cause of death, and chronic heart failure was frequent. However, sepsis was the most common cause of death within one year after bypass surgery.

**Table:** Table 2 Leading causes of death

Leading causes of death	All	Dialysis patient group		
	(n = 67)	(n = 46)	Within 1 year (n = 31)	From the first year on (n = 15)
Cardiovascular event, n (%)	19 (28%)	12 (26%)	7 (23%)	5 (33%)
Heart failure	15	10	7	3
Myocardial infarction	2	1	0	1
Arrhythmia (VT/VF)	2	1	0	1
Cerebrovascular event, n (%)	4 (6%)	3 (7%)	3 (10%)	0 (0%)
Bleeding	2	1	1	0
Infarction	2	2	2	0
Respiratory event, n (%)	6 (9%)	3 (7%)	1 (3%)	2 (13%)
Pneumonia	5	3	1	2
Asthma	1	0	0	0
Renal failure, n (%)	5 (7%)	5 (11%)	3 (10%)	2 (13%)
Gastrointestinal event, n (%)	2 (4%)	2 (4%)	2 (6%)	0 (0%)
Bleeding	1	1	1	0
Peritonitis	1	1	1	0
Pancreatitis/cholangitis, n (%)	1	0	0	0
Malignant disease, n (%)	5 (7%)	1 (2%)	1 (3%)	0
Sepsis, n (%)	14 (21%)	11 (24%)	9 (29%)	2 (13%)
Suicide, n (%)	1 (1%)	1 (2%)	1 (3%)	0
Unknown, n (%)	10 (15%)	8 (17%)	4 (13%)	4 (27%)

VT: ventricular tachycardia; VF: ventricular fibrillation

### Analyses of ROC curves

For survival rate, an ALC value of 1030/μL was calculated as the cut-off [sensitivity 53%, specificity 74%, the area under the curve (AUC) 0.64]. A value of 1260/μL (sensitivity 76%, specificity 52%, AUC 0.65) was considered the cut-off value for limb salvage. In the dialysis patient group, 1030/μL (sensitivity 61%, specificity 56%, AUC 0.55) was considered the cut-off survival value. A value of 1170/μL (sensitivity 92%, specificity 48%, AUC 0.67) was considered the cut-off value for limb salvage.

### Kaplan–Meier analysis

**Figure 1:**
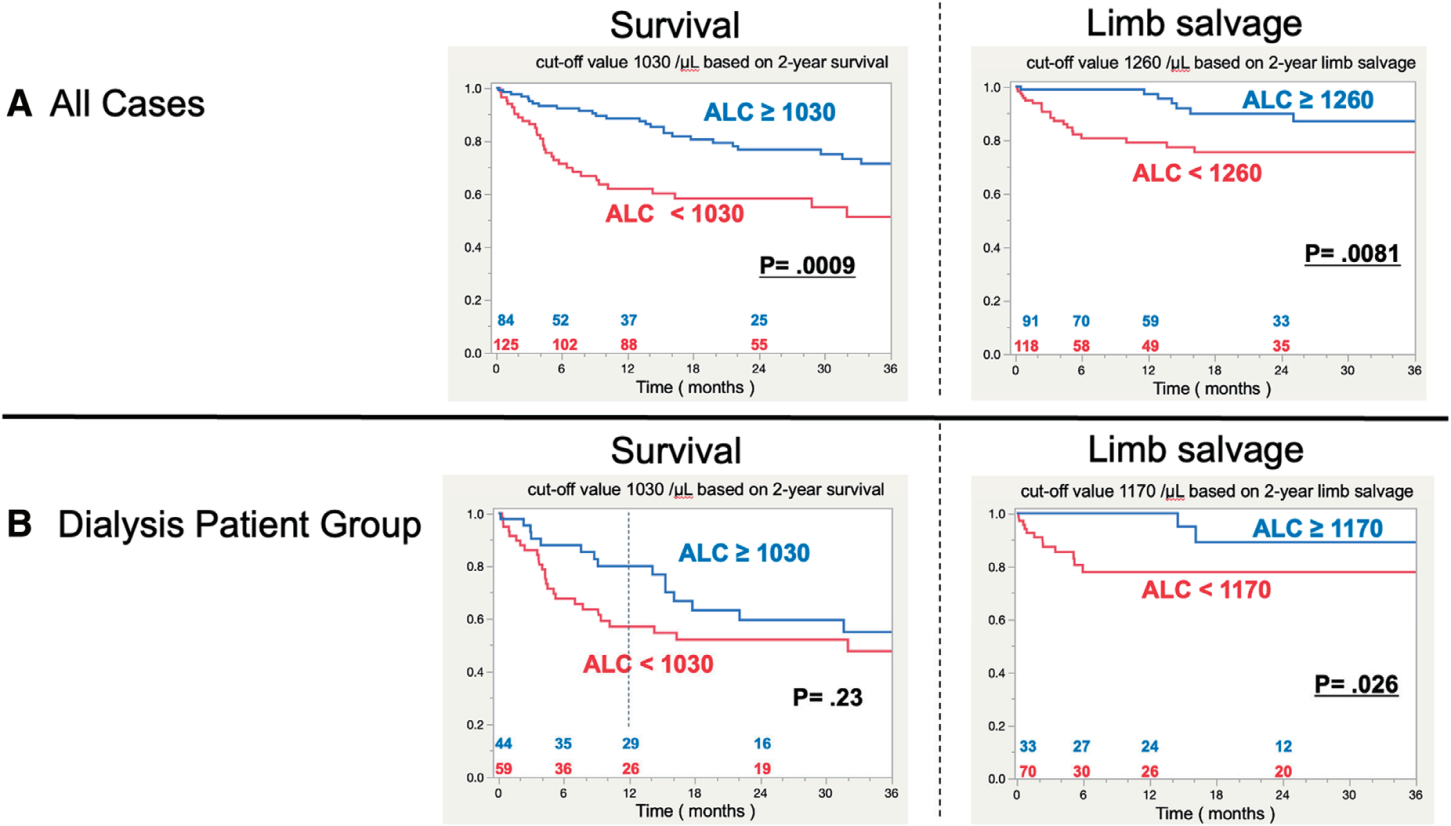
Fig. 1 Kaplan–Meier curves for overall survival and limb salvage. (**A**) All cases and (**B**) the dialysis patient group. ALC: absolute lymphocyte count (/μL)

The survival rate of the higher ALC group was significantly higher than that of the lower ALC group (2-year survival rate: 76.6% vs. 58.2%, p = 0.0009) (**Fig. 1A**). The mean ± SD of ALC was 1445 ± 704/μL in the patients who survived more than 2 years and 1124 ± 554/μL in the patients who died within 2 years (p = 0.0063).

Although there was no significant difference in primary and secondary graft patency between the higher and lower ALC groups (2-year graft patency rate: 63.9% vs. 63.6%, p = 0.73, and 76.8% vs. 75.8%, p = 0.73, respectively), limb salvage of the higher ALC group was significantly higher than that of the lower ALC group (2-year limb salvage rate: 89.8% vs. 75.5%, p = 0.0081) (**Fig. 1A**). The mean ± SD of ALC was 1416 ± 663/μL in patients who avoided major amputation for over 2 years and 1126 ± 628/μL in patients who underwent major amputation within 2 years (p = 0.032).

Among dialysis patients, the one-year survival rate was higher in the higher ALC group than in the lower ALC group. Still, no significant difference was observed between the two groups divided as per the cut-off value (1-year survival rate: 80.0% vs. 57.0%, and 2-year survival rate: 59.5% vs. 52.0%, p = 0.23) (**Fig. 1B**). The limb salvage rate of the higher ALC group was significantly higher than that of the lower ALC group (2-year limb salvage rate: 89.1% vs. 75.7%, p = 0.026) (**Fig. 1B**).

### Statistical analyses with Cox methods

Univariate analysis revealed that at age 80 or over, dyslipidemia, coronary artery disease, chronic heart failure, hemodialysis, nonambulatory status, low ALC, low hemoglobin, and low serum albumin were all significantly correlated with mortality (**Table 3**). Multivariate analysis revealed that the factors independently associated with mortality in all cases were age 80 or over, which are dyslipidemia, coronary artery disease, hemodialysis, and low serum albumin.

**Table:** Table 3 Univariate and multivariate analyses (Cox regression) for mortality (all cases)

Variable	Univariate analysis			Multivariate analysis		
	p	HR	95% CI	p	HR	95% CI
High age (≥80 years)	0.046	1.82	1.01–3.26	0.020	2.10	1.123–3.90
Male	0.31	1.34	0.76–2.36			
Low body mass index (<18.5 kg/m^2^)	0.23	1.40	0.81–2.43			
Hypertension	0.11	1.72	0.88–3.37			
Dyslipidemia	0.026	0.53	0.30–0.93	0.020	0.50	0.28–0.90
Diabetes mellitus	0.12	1.66	0.87–3.17			
Coronary artery disease	0.0012	2.26	1.38–3.69	0.0002	2.68	1.59–4.54
Chronic heart failure	0.011	2.24	1.20–4.16	0.48	1.27	0.65–2.46
Cerebrovascular disease	0.13	1.50	0.88–2.54			
Hemodialysis	<0.0001	2.84	1.69–4.78	0.0034	2.44	1.34–4.42
Nonambulatory	0.0061	2.04	1.23–3.39	0.23	1.40	0.80–2.46
WIfI grade 4	0.13	1.66	0.87–3.17			
Contralateral limb status CLTI	0.088	1.52	0.94–2.45			
Low ALC (<1030/μL)	0.0012	2.23	1.37–3.62	0.50	1.21	0.69–2.11
Low hemoglobin (<10 g/dL)	0.0027	2.09	1.29–3.39	0.81	0.93	0.53–1.66
Low serum albumin (<3.0 g/dL)	0.0007	2.30	1.42–3.74	0.0075	2.12	1.22–3.67

WIfI: Wound, Ischemia, and foot Infection; CLTI: chronic limb-threatening ischemia; ALC: absolute lymphocyte count

A univariate analysis revealed that low body mass index, nonambulatory status, low ALC, low hemoglobin, and low serum albumin were significantly correlated with limb loss in all cases (**Table 4**). Multivariate analysis revealed that the factor independently associated with limb loss in all cases was low ALC.

**Table:** Table 4 Univariate and multivariate analysis (Cox regression) for limb loss (all cases)

Variable	Univariate analysis			Multivariate analysis		
	p	HR	95% CI	p	HR	95% CI
High age (≥80 years)	0.82	1.12	0.42–2.95			
Male	0.53	1.27	0.37–1.68			
Low body mass index (<18.5 kg/m^2^)	0.039	2.23	1.04–4.79	0.083	1.98	0.91–4.27
Hypertension	0.40	1.40	0.33–1.56			
Dyslipidemia	0.48	1.33	0.34–1.65			
Diabetes mellitus	0.88	1.07	0.46–2.50			
Coronary artery disease	0.12	1.78	0.86–3.69			
Chronic heart failure	0.42	1.50	0.57–3.95			
Cerebrovascular disease	0.096	1.88	0.89–3.98			
Hemodialysis	0.40	1.36	0.66–2.79			
Nonambulatory	0.034	2.31	1.07–4.99	0.35	1.49	0.64–3.45
WIfI grade 4	0.29	1.68	0.64–4.39			
Contralateral limb status CLTI	0.065	1.98	0.96–4.09			
Low ALC (<1260/μL)	0.011	2.87	1.27–6.48	0.044	2.38	1.03–5.55
Low hemoglobin (<10 g/dL)	0.029	2.23	1.09–4.58	0.52	1.31	0.57–2.98
Low serum albumin (<3.0 g/dL)	0.011	2.56	1.24–5.29	0.20	1.71	0.75–3.90

WIfI: Wound, Ischemia, and foot Infection; CLTI: chronic limb-threatening ischemia; ALC: absolute lymphocyte count

## Discussion

One of the most significant differences between Japan and other countries is the high ratio of patients undergoing hemodialysis among patients with CLTI. In Japan, risk factors for all-cause mortality in patients undergoing revascularization for CLTI included old age, impaired mobility, low body mass index, renal failure, heart failure, and high WIfI (Wound, Ischemia, and foot Infection) grade, based on the SPINACH study.^7)^ Miyata et al. proposed a risk prediction model for outcomes of revascularization for CLTI based on the JAPAN Critical Limb Ischemia Database (JCLIMB), and hemodialysis was a strong predictor of major amputation and mortality.^8)^ Hemodialysis is a common, primary risk factor for mortality and limb loss. Still, risk factors in the dialysis group have yet to be fully clarified, whereas patients undergoing hemodialysis have different prognoses. Predictability of prognosis in patients undergoing hemodialysis is also essential for CLTI treatment strategy in Japan.

We searched for an accurate and simple indicator to predict the prognoses of patients, including those undergoing hemodialysis. ALC reflects immunological capacity and nutritional status over a certain period. Although neutrophil counts fluctuate considerably due to acute inflammation and infection, ALC is not susceptible to these factors in the short term. Multi-item nutritional evaluation measures are usually too complex for regular use in clinical practice, but ALC is readily available from routine blood tests. We think that ALC may be the simplest and most readily evaluated variable that reflects patient nutritional and immune status. We suggest that ALC is helpful in predicting the life prognosis of patients with chronic heart failure and malignant diseases.^16^^,^^17)^ However, another item, such as serum albumin, has been adopted as representative of the nutrition index,^9)^ and few reports are available on ALC in CLTI treatment. Therefore, we focused on ALC in this study.

ALC differed significantly between dialysis and nondialysis patient groups, and we investigated outcomes in all cases and in the dialysis patient group. The optimal cut-off value of ALC was unknown because it fluctuates according to the study population and endpoint. Patients with CLTI tend to have lower ALC than the general population because of their poor nutritional and immune status, and optimal cut-off values of ALC were assumed to differ. Thus, in this study, we found respective cut-off values of ALC on survival and limb salvage rates in each group.

The lower ALC group showed poorer survival and poorer limb salvage than the higher ALC group, and these results suggest that ALC helps predict life and limb prognosis after infrainguinal bypass surgery in patients with CLTI. However, hemodialysis was one of the factors independently associated with mortality, and it is difficult but essential to predict the life prognosis of patients undergoing hemodialysis. The one-year survival rate in the dialysis patient group was higher in the higher ALC group than in the lower ALC group. Sepsis and heart failure were the primary causes of death within one year after bypass surgery in the dialysis patient group.

ALC reflects a general immune state closely related to infection.^18)^ Many studies have suggested that nutritional state is highly correlated with cardiovascular events, including heart failure,^19^^,^^20)^ and ALC is suggested as a predictor of life prognosis of patients with chronic heart failure.^16)^ Therefore, ALC may stratify the risk of sepsis and heart failure. Because patients undergoing hemodialysis had rather poor prognoses and 31 patients (30%) died within one year, we referred to the 1-year survival and limb salvage. In the dialysis patient group, ALC may be helpful in predicting prognosis within one year after surgery. There are few reports on the outcomes of bypass surgery for CLTI in the dialysis patient group. Accordingly, we think that the findings in the dialysis patient group are worth reporting. Further research is needed with an adequate number of dialysis patients. Regarding limb salvage, low ALC was the only factor independently associated with limb loss, and this result suggests that ALC may be a strong predictor of limb salvage after bypass surgery, independent of hemodialysis. Uncontrollable infection was the most frequent reason for major amputation. ALC can stratify the risk of limb-threatening infection even in the dialysis patient group.

This study had several limitations. First, it was carried out at a single center. Second, it was a retrospective study. There was also a selection bias. An optimal cutoff value of ALC has yet to be established, and finally, the sample size needs to be more robust for multivariate analysis in the dialysis patient group. The prevalence of dyslipidemia was low among patients with CLTI, as in other reports from Japan, and the proportion of patients who received statins was low.^8^^,^^21)^ We think that the low proportion of statins may influence cardiovascular events and prognoses. We could not examine outcomes after endovascular treatment because there were few patients who underwent endovascular treatment alone. Thus, further research on endovascular treatment and bypass surgery groups is required to clarify ALC's usefulness in developing a CLTI strategy. However, ALC can potentially improve prognosis accuracy in treatment for CLTI because ALC accurately and sensitively reflects patient life- and limb-threatening conditions. ALC has not been regularly evaluated in the treatment of CLTI, but we think that it is worth adding to the analysis as a potential indicator in clinical use. We think that minimally invasive endovascular treatment, primary major amputation, or even nonsurgical treatment may be an option instead of bypass surgery for patients with low ALC because they are expected to have poor prognoses. On the contrary, we think that active bypass surgery is an appropriate option for patients with high ALC because they are expected to have good prognoses. We believe that a study like this one, but including a large number of patients undergoing hemodialysis, may improve the predictability of prognosis in treating CLTI.

## Conclusion

ALC appears promising as a predictive indicator after bypass surgery in the treatment of CLTI. In particular, ALC is expected to help predict limb prognosis after bypass surgery in hemodialysis patients. ALC reflects the patient's nutritional and immune state and may stratify the risk of infection and heart failure, which are closely related to life- and limb-threatening conditions. ALC may help improve the predictability of prognosis in CLTI treatment.

## Acknowledgments

We thank Dr. Yohei Kawasaki and Dr. Yuki Shiko at the Research Administration Center of Saitama Medical University for advising us on statistical analysis.

## Disclosure Statement

All authors have no conflicts of interest.

## Author Contributions

Study conception: SY, TH, and JD

Data collection: all authors

Analysis: SY and TH

Investigation: SY, TH, and JD

Manuscript preparation: SY and TH

Funding acquisition: None

Critical review and revision: all authors

Final approval of the article: all authors

Accountability for all aspects of the work: all authors.
